# Higher blood glucose and larger fluctuations detected postoperatively using continuous glucose monitoring: a preliminary study following total knee or hip arthroplasty

**DOI:** 10.1186/s40634-019-0181-9

**Published:** 2019-04-01

**Authors:** Yuki Maeda, Nobuo Nakamura, Takashi Tsujimoto, Nobuhiko Sugano

**Affiliations:** 1 Department of Orthopaedic Medical Engineering, Osaka University Graduate School of Medicine, 2-2 Yamadaoka Suita, Osaka, 565-0871 Japan; 2 Center of Arthroplasty, Kyowakai Hospital, Suita, Japan

**Keywords:** Total joint arthroplasty, Continuous glucose monitoring, Glycemic variability, Periprosthetic joint infection

## Abstract

**Background:**

The control of diabetes mellitus (DM) should help reduce the incidence of periprosthetic joint infection (PJI). Self-monitoring of blood glucose (SMBG) concentration is usually undertaken at fixed time-points. Therefore, the extent of postoperative blood glucose fluctuation might be underestimated. To provide a more comprehensive assessment, continuous glucose monitoring (CGM) is beginning to be used. However, no previous studies have evaluated blood glucose concentrations using CGM following orthopedic surgery. Therefore, the differences between the maximum blood glucose concentrations measured using SMBG and CGM, and the mean amplitude of the glycemic fluctuation in patients with frank diabetes mellitus (DM) or pre-diabetes were evaluated.

Blood glucose was measured in 20 patients who had undergone total hip or total knee arthroplasty (12 patients with DM and eight with pre-diabetes). Patients were fitted with a CGM device in the operating room, which was worn for 6 days postoperatively, and used to evaluate blood glucose concentration continuously. SMBG was performed simultaneously for the same period.

**Results:**

The mean difference between the maximum blood glucose concentrations measured using SMBG and CGM was 25.0 ± 20.3 mg/dl (range, − 17 to 81 mg/dl), with the concentrations measured using CGM tending to be higher than those measured using SMBG (*P* = 0.04). Blood glucose concentrations measured using CGM tended to be higher than those measured using SMBG until postoperative day 2, and to decrease gradually after postoperative day 4. There were no significant differences in the standard deviation of the blood glucose concentrations between the two groups.

**Conclusions:**

Blood glucose concentrations > 200 mg/dl and larger fluctuations were more frequently recorded using CGM than SMBG, especially until postoperative day 2. Thus, CGM is more useful for the identification of high blood glucose concentrations and larger fluctuations. However, this information was not provided in real time.

## Background

Patient factors involved in the etiology of periprosthetic joint infection (PJI) have frequently been reported, and include a history of diabetes mellitus (DM) (Kunutsor et al., 2016). Control of DM helps reduce the incidence of PJI. To evaluate DM pre-operatively, hemoglobin (Hb) A1c levels can be used, and postoperatively, blood glucose concentrations are used clinically. Previous studies have shown that the incidence of infection is higher when blood glucose concentrations are > 200 mg/dl postoperatively (Mraovic et al., 2011; Stryker et al., 2013; Tarabichi et al., 2017). Blood glucose concentrations are normally measured at set time points by self-monitoring (SMBG) using a glucometer, but this method does not enable the continuous assessment of postoperative blood glucose levels. To enable such an assessment, continuous glucose monitoring (CGM) is beginning to be used (Boom et al., 2014; Danne et al., 2017; Keenan et al., 2009), which more readily facilitates the identification of blood glucose variations. Indeed, blood glucose concentrations obtained using CGM sometimes show abnormal fluctuations or hypoglycemia, even when the values obtained using SMBG are normal (Danne et al., 2017).

A number of studies have been conducted to date in which blood glucose has been evaluated using CGM (Madhu et al., 2013; Rasbach et al., 2014; Zhou et al., 2017), but none have evaluated the variability in postoperative blood glucose concentrations using CGM following orthopedic surgery.

The aim of this study was to use CGM to evaluate the postoperative variability in blood glucose concentrations in patients who had undergone either total knee arthroplasty (TKA) or total hip arthroplasty (THA), and to evaluate the differences between the maximum blood glucose concentrations measured by SMBG and by CGM, and the mean amplitude of the glycemic fluctuation in patients with frank DM or pre-diabetes.

## Methods

This prospective observational study was approved by our institutional review board (Kyowakai Research Ethics Board #16–02). Written informed consent was obtained from all the participants. Three hundred patients who underwent THA or TKA in our hospital between October 2016 and September 2017 were screened. When a patient’s fasting blood glucose was in the range 110–126 mg/dl, and if they did not have a history of DM, an oral glucose tolerance test (OGTT) was performed. Pre-diabetes (borderline DM) was defined by a blood glucose of 140–199 mg/dl, while DM was defined by a blood glucose of > 200 mg/dl during the OGTT (American Diabetes Association, 2017).

Twenty-six patients were diagnosed with DM or pre-diabetes among those who underwent THA or TKA. The blood glucose concentration of each patient with DM was controlled using rapid-acting insulin and/or long-lasting insulin before surgery, under the supervision of a diabetologist. Patients classified as pre-diabetic did not receive any treatment.

A CGM system comprising a soft sensor (Enlite sensor; Medtronic Minnesota, USA) and a small, light and water-proof recorder (iPro 2, Medtronic, Minnesota, USA) was used. Patients were fitted with the CGM device in their abdominal region, and subcutaneous glucose concentrations were recorded every 5 min for 6 days postoperatively. Caplin et al. (Caplin et al., 2003) have reported that the mean blood glucose concentration is 2.3 mg/dl higher than the mean subcutaneous concentration. However, subcutaneous glucose concentrations correlate closely with the equivalent blood glucose concentrations. Therefore, blood glucose concentrations were also measured regularly using SMBG to adjust the subcutaneous glucose values, and the corrected blood glucose values were subsequently evaluated.

First, the differences between the maximum blood glucose levels measured using SMBG and CGM, and the number of patients who recorded blood glucose concentrations > 200 mg/dl using both CGM and SMBG, were established. Two patients demonstrated substantial fluctuations in their postoperative blood glucose concentrations. Second, the mean amplitude of the glycemic fluctuations was evaluated using the standard deviations of the recorded values (Nishimura et al., 2008). The standard deviations of the blood glucose concentrations were also compared between the diabetic and pre-diabetic patients, and the relationships between the pre-operative HbA1c values and the glycemic fluctuations on each day following surgery were assessed.

All statistical analyses were performed using Bell Curve Ekuseru-Toukei (Social Survey Research Information Co., Ltd., Tokyo, Japan). Differences between the groups were evaluated using the paired *t*-test, Fisher’s Exact test and relationships between variables were evaluated using multiple regression analysis. Statistical significance was accepted when *P* < 0.05.

## Results

Blood glucose concentrations could be measured for > 5 days in 20 of the 26 patients (5 men and 15 women; mean age, 70.2 years). The mean pre-operative HbA1c was 6.7 ± 1.1%, and the mean pre-operative fasting blood glucose was 130.9 ± 35.8 mg/dl. Of these 20 patients, 12 were diabetic and eight were pre-diabetic. There was no significant difference in any parameter between the two groups (Table 1).

**Table 1:** Characteristics of the diabetic and prediabetic groups

	Age (years)	Sex (n)	Body mass index (kg/m^2^)	Surgery (n)	Fasting blood glucose (mg/dl)	HbA1c (%)
Diabetic group (*n* = 12)	70.4 ± 5.4	Male 4 Female 8	26.6 ± 4.2	THA 8 TKA 4	141.4 ± 40.5	7.0 ± 1.2
Pre-diabetic group (*n* = 8)	68.3 ± 8.6	Male 1 Female 7	25.4 ± 4.1	THA 3 TKA 5	125.8 ± 16.5	6.2 ± 0.6
*P* value	0.3	0.30*	0.5	0.21*	0.61	0.08

Data are mean ± SD or n number. * Fisher’s Exact test used. SD, standard deviation; THA, total hip arthroplasty; TKA, total knee arthroplasty; HbA1c, glycated hemoglobin

The mean difference between the maximum blood glucose concentrations measured using SMBG and CGM was 25.0 ± 20.3 mg/dl (range, − 17 to 81 mg/dl), with the concentrations measured using CGM tending to be higher than those measured using SMBG (paired *t*-test; *P* = 0.04) The maximum blood glucose concentrations measured using CGM were > 200 mg/dl in 16 patients (80%) and those measured using SMBG were > 200 mg/dl in 11 patients (55%).

**Fig. 1:**
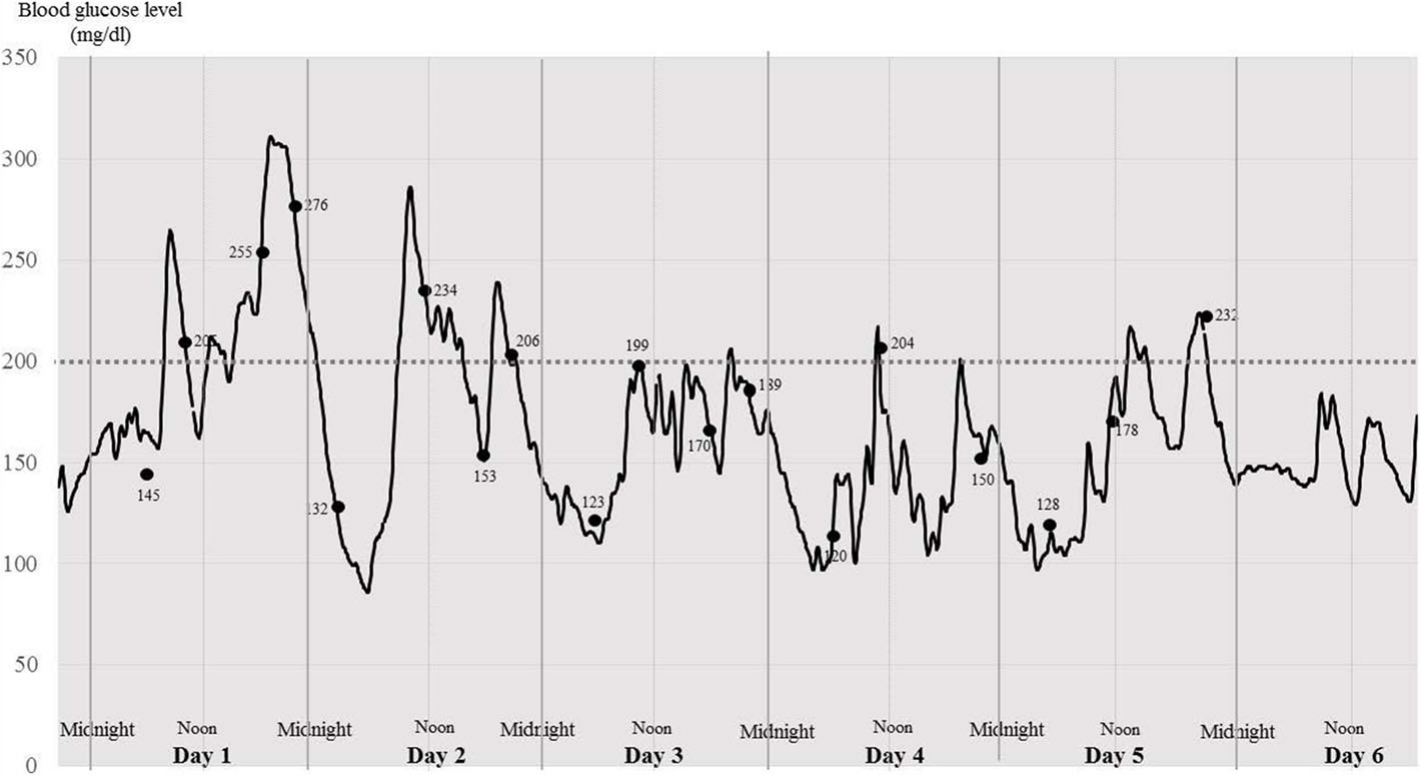
Continuous blood glucose trace for Patient 1. Postoperative blood glucose concentrations in an 81-year-old woman with pre-diabetes in whom a bilateral total knee arthroplasty (TKA) had been performed. X-axis, postoperative day; Y-axis, blood glucose concentration (mg/dl). Black spots, blood glucose concentrations measured using self-monitoring of blood glucose (SMBG). Each number is the blood glucose concentration (mg/dl) at that time point

**Fig. 2:**
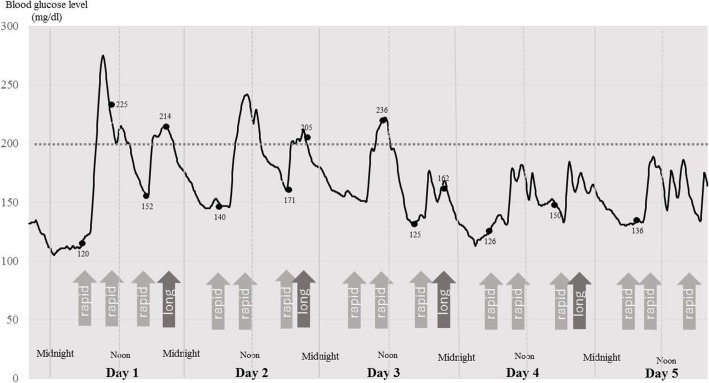
Continuous blood glucose trace for Patient 2. Postoperative blood glucose in a 73-year-old woman with diabetes in whom a right total hip arthroplasty (THA) had been performed. X-axis, postoperative day; Y-axis, blood glucose concentration (mg/dl). Black spots, blood glucose concentrations measured using SMGB. Each number is the blood glucose concentration (mg/dl) at that time point. Arrow (rapid), the administration of rapid-acting insulin; arrow (long), the administration of long-acting insulin

Typical variations in blood glucose concentration are shown, in a patient with prediabetes and in another with DM, in order to clearly demonstrate the output of CGM. Patient 1 was an 81-year-old female pre-diabetic patient who underwent TKA, and whose postoperative blood glucose concentrations are shown in Fig. 1. Patient 2 was a 73-year-old female diabetic patient who underwent THA, and whose postoperative blood glucose concentrations are shown in Fig. 2. Patient 2 administered rapid-acting and long-lasting insulin before and after surgery. Figure 1 shows that for Patient 1 the fluctuations in blood glucose tended to be larger until postoperative day 2, after which they tended to decrease gradually, without the administration of insulin, which typical of the study cohort. The peak blood glucose concentrations measured using CGM in the postoperative period were higher than those measured using SMBG. The example graph for Patient 2 shows that the fluctuation in blood glucose tended to be large until postoperative day 2, despite the administration of insulin.

The mean amplitudes of the glycemic fluctuation are shown in Table 2. The standard deviations of the blood glucose concentrations tended to be large until postoperative day 2 and then decreased gradually after postoperative day 4. There was no difference in the standard deviations between the diabetic and pre-diabetic groups (Table 2). Furthermore, the standard deviations over postoperative days 1–5 did not correlate with HbA1c (correlation coefficient, *r* = − 0.089, 0.081, − 0.15, 0.251, and − 0.047, respectively).

**Table 2:** Mean amplitude of the fluctuation in glucose concentration in the diabetic and pre-diabetic groups

	Day 1	Day 2	Day 3	Day 4	Day 5
All cases (*n* = 20)	32.7	26.6	21.6	21.5	17.4
Diabetic group (*n* = 12)	35.8	27.7	17.1	21.5	15.9
Pre-diabetic group (*n* = 8)	28.8	25.1	27.3	21.4	19.3
*P* value	0.35	0.65	0.07	0.99	0.23

Data are the standard deviation of the subcutaneous glucose concentration on each post-operative day (mg/dl)

## Discussion

Many studies have shown that DM is a risk factor for PJI (Kunutsor et al., 2016; Jämsen E, et al. 2012; Sato et al., 2017; Schierenbeck et al., 2017) and have identified associations between the development of postoperative infections and high HbA1c (Mraovic et al., 2011; Stryker et al., 2013; Tarabichi et al., 2017). In addition, others have identified perioperative hyperglycemia as a risk factor following total joint arthroplasty (Mraovic et al., 2011; Stryker et al., 2013). Mraovic et al. showed that the incidence of infection doubled when blood glucose concentrations before breakfast were > 200 mg/dl (Mraovic et al., 2011), and Stryker et al. reported that it was 3.75 times higher when the mean post-operative blood glucose was > 200 mg/dl (Stryker et al., 2013). Thus, it is important to measure and control blood glucose concentration in the perioperative period.

However, blood glucose concentrations tend to be measured only at fixed time points, usually three or four times a day at most. Thus, the extent of postoperative blood glucose fluctuation might frequently be underestimated, which motivated us to assess postoperative blood glucose concentrations continuously following TKA and THA. In the present study, blood glucose concentrations measured using CGM were higher than those measured using SMBG, and showed large fluctuations, especially until postoperative day 2. The mean amplitude of the glycemic fluctuations was not significantly different between patients with diabetes and those with pre-diabetes, and the fluctuation in glycemia did not correlate with the pre-operative HbA1c.

According to the guidelines for the prevention of surgical site infection (SSI), a blood glucose > 200 mg/dl in the first 48 h following surgery is associated with a higher risk of SSI (O'Hara et al., 2018). Moreover, a blood glucose concentration < 200 mg/dl is recommended to reduce the likelihood of infection (Mraovic et al., 2011; Stryker et al., 2013). In our study, postoperative blood glucose concentrations measured using CGM reached > 200 mg/dl in 80% of the participants, but these levels were detected less frequently using SMBG. However, the blood glucose values obtained using CGM were not provided in real time. Therefore, frequent monitoring of blood glucose concentrations obtained using SMBG is currently recommended until at least postoperative day 2, not only for diabetic patients, but also for patients with pre-diabetes.

The mean amplitude of the glycemic fluctuations was not significantly different between the pre-diabetic and diabetic groups. Large fluctuations in blood glucose may be an important predictor of mortality, as well as of major adverse cardiac events (Su et al., 2013). Although no previous publications have suggested that patients with pre-diabetes may be at greater risk of postoperative complications, including infections, our findings imply that pre-diabetic patients should be as vigilant regarding postoperative complications as diabetic patients.

We hypothesized that HbA1c level can be used as a marker of glycemic control, and is routinely measured in many patients with diabetes (Rohlfing et al., 2002). Hwang demonstrated a strong correlation between preoperative HbA1c and postoperative glucose concentrations in patients who underwent TKA (Hwang et al., 2015). However, in our study, the degree of postoperative blood glucose fluctuation did not correlate with HbA1c. Consistent with this, Suwa et al. reported that the HbA1c value did not reflect glucose fluctuation as well as the short-term mean glucose value in 43 diabetic patients (Suwa et al., 2010). Thus, other, yet to be identified, glycemic markers may prove to be more useful than HbA1c for the evaluation of fluctuations in blood glucose (Suwa et al., 2010; Shohat et al., 2017).

There were several limitations to this study. First, multivariate analysis was not performed because of the small number of participants, and for the same reason the statistical power was low for the comparison of the mean amplitude of the glycemic fluctuations between the pre-diabetic and diabetic groups. Thus, further evaluation using a larger number of patients is required. Second, blood glucose data obtained using the CGM system in this study could not provide a real-time output, such that hypoglycemia could not be rapidly detected, and blood glucose could not be measured after the removal of the CGM device. Finally, we did not evaluate the blood glucose concentrations of patients who developed infections or other complications following TJA. However, CGM was found to be easy to use and a useful tool for the continuous measurement of blood glucose concentrations and their fluctuation, which could not be achieved using SMBG. If the blood glucose fluctuations in patients who subsequently developed infections could be assessed, and sufficient data for such an analysis obtained, this would be helpful to define the ideal blood glucose range to minimize the risk of PJI.

## Conclusions

We conclude that higher blood glucose concentrations and larger fluctuations are detected using CGM than SMBG, and that higher blood glucose concentrations exist postoperatively than were previously thought, especially during days 1–2. Thus, CGM may represent a useful tool for the prevention of extreme blood glucose concentrations, which should help to reduce the risk of PJI.
